# Limb Salvage Surgery for Ponatinib-Induced Bilateral Chronic Limb-Threatening Ischemia in a Patient with Chronic Myeloid Leukemia with T315I Mutation in BCR-ABL: A Case Report

**DOI:** 10.3400/avd.cr.23-00059

**Published:** 2023-11-28

**Authors:** Takuo Nomura, Akito Hata

**Affiliations:** 1 Department of Vascular Surgery, Juzen Memorial Hospital, Hamamatsu, Shizuoka, Japan; 2 Division of Thoracic Oncology, Kobe Minimally Invasive Cancer Center, Kobe, Hyogo, Japan

**Keywords:** limb salvage, ponatinib, chronic limb-threatening ischemia

## Abstract

A 72-year-old woman with chronic myeloid leukemia with T315I mutation in breakpoint cluster region-abelson (BCR-ABL) was treated with ponatinib. During the course of her treatment, chronic limb-threatening ischemia developed in both lower extremities, and the left lower extremity was amputated below the knee at a previous hospital. She was referred to our department for salvage of the right lower extremity. We performed a foot bypass and multidisciplinary treatment of the wound, and achieved epithelialization in about 1 month. The rate of vascular occlusive events with ponatinib has been reported to be high, and we believe that careful monitoring is important during use.

## Introduction

Chronic limb-threatening ischemia (CLTI) is a general term used to describe conditions of the lower limb that are at risk of amputation and require immediate therapeutic intervention, such as lower limb ischemia, tissue loss, neuropathy, and infection.^1)^ In Japan, it is reported that approximately 70%–80% of CLTIs occur in diabetics and 50% in hemodialysis patients. CLTI is mostly caused by atherosclerotic chronic arterial disease.^2)^ Other causes of limb ischemia are mainly trauma, non-atherosclerotic chronic arterial diseases such as vasculitis and Buerger’s disease, and drug-induced CLTI seeming to be relatively rare.^3)^

Tyrosine kinase inhibitors (TKIs) have dramatically improved the prognosis of patients with chronic myeloid leukemia (CML). The improvement in outcomes has been accompanied by a prolonged duration of TKI administration, and the occurrence of cardiovascular events (CVEs) related to TKIs has increased.^4)^ Ponatinib is a third-generation TKI, with a sensitivity to T315I mutation in BCR-ABL, and widely used after failure of other TKIs such as imatinib, whose resistant mechanism is mainly T315I mutation.^5)^ High incidence of CVE was demonstrated in clinical trials,^6)^ but occurrence of CLTI was scarcely reported.

We here report a CML case of ponatinib-induced CLTI in which bilateral major amputations of the lower extremities were avoided by limb salvage surgery.

## Case Report

A 72-year-old woman was diagnosed with CML after a bone marrow examination for an increased white blood cell count showed a Philadelphia chromosome, and dasatinib, a second-generation TKI, was initiated as the first-line treatment. Two years later, dasatinib was switched to imatinib, a first-generation TKI, because of a possible drug-induced heart failure. Five years after switching to imatinib, white blood cell and platelet counts were elevated with increased juvenile granulocytes. Genetic analysis revealed a T315I mutation in BCR-ABL, and imatinib was switched to third-generation ponatinib. The initial dose was reduced to 15 mg/day because of her elderly age. Since hematologic response was successful but molecular biological response was poor, the dose was increased to 30 mg/day. The previous hematologist only performed an echocardiogram and electrocardiogram (ECG) before administering ponatinib and did not perform an ankle brachial index (ABI) because her dorsalis pedis artery was palpable. They did not administer antiplatelet drugs for CVE prophylaxis because she had no atherosclerotic risk factors such as diabetes or hypertension and her cardiac function tests were normal.

One year after starting ponatinib, she was admitted to the previous hospital because of deterioration of color tone and ulceration of both lower extremities. Angiographic findings showed multiple arterial occlusions below the knees in both lower extremities. Her previous hematologist and vascular surgeon investigated the cause of ischemia in both lower extremities. After ruling out autoimmune disease and acute arterial occlusive disease based on clinical findings and blood tests, they diagnosed ponatinib-induced CLTI. Her left lower extremity with severe necrosis was treated, but the wound became infected and was amputated below the knee.

**Figure 1:**
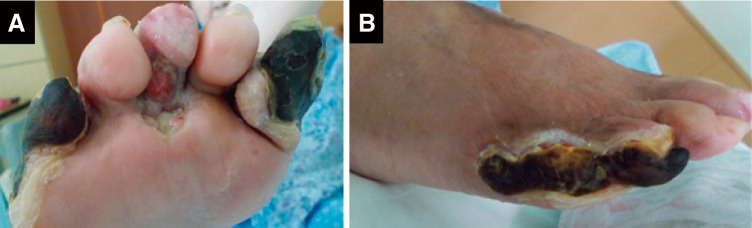
Fig. 1 Wound condition of the lower extremity at the time of admission. (**A**) The first and fifth toes had necrosis of the entire toe. The third toe showed deep ulceration, and ulceration formed on the medial side of the fourth toe. (**B**) Necrosis had spread to the lateral side of the fifth toe.

**Figure 2:**
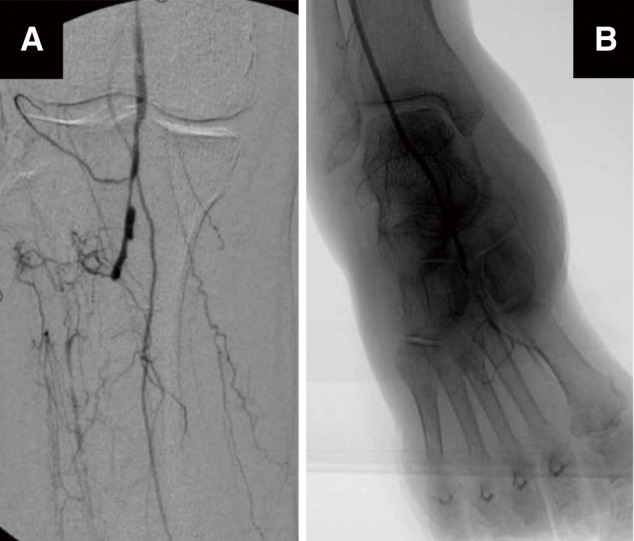
Fig. 2 Preoperative angiography. (**A**) Severe stenosis of the popliteal artery was observed. The anterior tibial, posterior tibial, and peroneal arteries were occluded. (**B**) The distal anterior tibial artery and dorsalis pedis artery were contrasted via collateral vessels.

**Figure 3:**
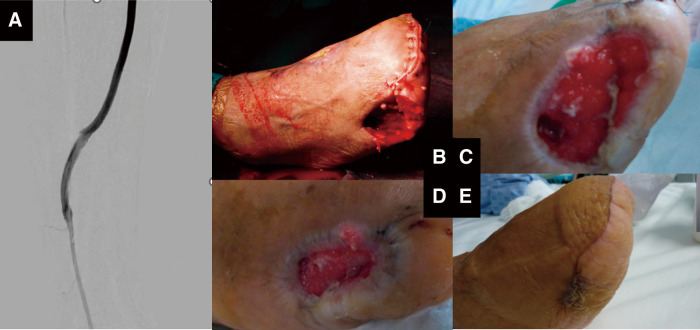
Fig. 3 Postoperative angiography and wound healing process. (**A**) Bypass graft was in good patency. (**B**) The forefoot wound could be closed but not the lateral side of the fifth toe. (**C**) Two weeks after surgery, the wound edge had not shrunk. (**D**) Wound reduction rate of 50% was achieved 2 weeks after starting LDLA. (**E**) Epithelialization was achieved after 1 month of LDLA. LDLA: low-density lipoprotein apheresis

Her right lower extremity showed progressive necrosis (**Figs. 1A** **and** **1B**), and she was referred to our department for limb salvage. The transcutaneous oxygen pressure in the right lower extremity was 27 mmHg in the dorsal foot region and 32 mmHg in the plantar region. Angiography showed severe stenosis of the right popliteal artery and occlusion of all arteries below the knee (**Fig. 2A**). On the other hand, blood flow below the ankle joint was well distributed (**Fig. 2B**). Since CML was stable in the chronic phase, we chose bypass surgery for revascularization. Surgery was performed under general anesthesia, and the great saphenous vein was first taken from the right lower extremity. Next, the proximal anastomosis was identified as the middle portion of the superficial femoral artery (SFA) and the distal anastomosis was identified as the distal portion of anterior tibial artery (ATA). Heparin 3000 units was administered and the ACT was controlled for at least 200 seconds. The great saphenous vein was reversed and bypassed from SFA to ATA. Lesion findings and operative details were similar to those of usual atherosclerotic disease. Intraoperative angiography was performed to confirm good flow to the foot (**Fig. 3A**).

On the 14th postoperative day, transcutaneous oxygen pressure was tested, and the dorsal foot region improved to 74 mmHg and the plantar foot region to 64 mmHg. Debridement of the foot necrotic tissue was performed on postoperative day 15. Contaminated tissue was excised and wound irrigated with adequate saline solution. Only the lateral side of the fifth toe was open, and the wound was closed (**Fig. 3B**). The wound did not shrink despite 2 weeks of treatment for an open wound (**Fig. 3C**). We used low-density lipoprotein apheresis (LDLA) as adjuvant therapy. She was not a dialysis patient, so a dialysis catheter was placed, administrating LDLA for 2 hours twice a week. After a total of 4 sessions, the wound was reduced to half its size (**Fig. 3D**). Then, under the guidance of a physiotherapist, she began gait training on the parallel bars wearing footwear with her forefoot unloaded. Two weeks later, the wound was epithelialized (**Fig. 3E**). Physical therapy enabled her to live independently while using a wheelchair, and she was discharged home 65 days after surgery. The graft flow and leg wounds were fine, but 8 months later, she was admitted to another hospital due to a stroke.

## Discussion

The present case of ponatinib-induced CLTI was diagnosed by the previous hematologist and a vascular surgeon. Angiographic findings showed abrupt occlusion, which is also seen in Buerger’s disease. However, computed tomography showed calcified deposits in the arterial walls of the lower limbs, and furthermore, the patient’s age, gender, and lack of smoking history ruled out Buerger’s disease. The ECG was in sinus rhythm, there was no evidence of aortic atheroma, and thromboembolism was ruled out. She suddenly developed CLTI in both lower extremities only 1 year after starting ponatinib. Since her dorsalis pedis arteries were palpable before initiation, we consider ponatinib to be strongly associated as a cause of bilateral CLTI. However, the lack of regular ABI testing after initiation of medication by the previous physician was very disappointing.

It has already been shown in a number of observational studies that patients on TKIs develop CVEs with a high frequency.^4)^ Reports are particularly serious in patients receiving ponatinib, a third-generation TKI. In a phase I study with 33 months follow-up of ponatinib-treated patients, 37% had CVEs, 23% of which were considered severe.^5)^ In a phase II study (PACE study), arterial occlusion was observed in 19% of patients up to 12 months and in 29% up to 24 months.^6)^ TKIs are thought to cause CVE by inhibiting a number of off-targets and altering the function of cells involved in the development of CVE, such as vascular endothelial cells, platelets, macrophages, and mast cells.^7)^ In particular, ponatinib is thought to induce CVE by inhibiting vascular endothelial growth factor receptor,^8)^ causing hypertension, and by being involved in arterial spasm.^3)^ We believe that post-discharge cerebral infarction may also be caused by CVE. However, we cannot prove this.

For salvage of the ischemic foot, we must first perform revascularization. It is recommended that revascularization for CLTI should be selected based on the patient’s general condition, the severity of the foot, and the vascular lesion.^1)^ Taking these factors into account, we decided on bypass surgery. On the other hand, it has been reported that life expectancy of more than 2 years is also important in the selection of the procedure in cases of severe comorbidities,^9)^ and we discussed her prognosis. She had CML and a recent major lower limb amputation. As for CML, she had no anemia, low platelet counts, splenomegaly, and the presence of blasts in her peripheral blood. We also discussed the prognosis with her previous hematologist. Based on these results, we concluded that her CML was stable in the chronic phase and that she had a life expectancy of more than 2 years.

Iida et al. reported that bypass surgery is an unfavorable factor in patients with a history of major lower limb amputation.^2)^ In fact, we also considered endovascular treatment, but in Japan, there are limitations in the devise of below-knee lesions and problems with patency rates. In addition, given the patient’s background of imminent major amputation of both lower limbs due to ponatinib-induced CLTI, a secure salvage of the contralateral limb was the best hope for her and us. For these reasons, bypass surgery was considered the best option.

Wound management, which shortens wound healing time, is also important to start physical therapy early. In this case, the wound was partially open, resulting in delayed wound healing. We were able to achieve early epithelialization with LDLA and further adequate physical therapy. Kojima et al. reported 68.4% wound healing with LDLA in 19 cases of no-option CLTI considered non-responsive to revascularization.^10)^ They also reported that LDLA improved wound microcirculation by reducing blood viscosity.^10)^

We have learnt from this case about the existence of drug-induced CLTI and the importance of their prevention. As far as we could find in the literature, we could not find any case reports of ponatinib-induced CLTI. Before TKIs are used, patients should not only be assessed for cardiovascular risks such as diabetes, hypertension, and abnormal lipid metabolism but also cardiovascular monitoring such as ECG, echocardiography, and ABI should be performed. Even after use, we strongly recommend that regular monitoring should always be carried out, and if CVE is suspected, a cardiologist or cardiovascular surgeon should be consulted as soon as possible.

## Conclusion

We experienced a CML case of ponatinib-induced CLTI in both lower limbs, in which the risk of amputation of both lower limbs could be avoided by performing a limb salvage surgery. Among TKIs, ponatinib is associated with a particularly high incidence of CVE, so we strongly recommend cardiovascular risk assessment before use and regular monitoring of cardiovascular hemodynamics after use.

## Informed Consent

The patient involved in this case report gave her informed consent authorizing use and disclosure of her health information.

## Acknowledgments

We thank Mr. David Martin for English writing support.

## Disclosure Statement

Takuo Nomura declared no conflicts of interest. Akito Hata received lecture fees from Eli Lilly, Chugai, Astrazeneca, MSD, Taiho, and Boehringer Ingelheim and research fundings from Eli Lilly, Chugai, AstraZeneca, MSD, Taiho, and Boehringer Ingelheim.

## Author Contributions

Medical treatment: TN

Data collection: TN

Analysis: TN and AH

Investigation: TN

Manuscript preparation: TN and AH

Critical review and revision: all authors

Final approval of the article: all authors

Accountability for all aspects of the work: all authors.
